# Supplementary material for: Negotiation and honesty in artificial intelligence methods for the board game of Diplomacy
Source: Nat Commun. 2022 Dec 6;13:7214. doi: 10.1038/s41467-022-34473-5 (PMC9726752; doi:10.1038/s41467-022-34473-5)
Supplement: Supplementary file 1 — Supplementary Information [file 41467_2022_34473_MOESM1_ESM.pdf]

Supplementary Material: Negotiation and Honesty in Artificial Intelligence Methods for the Board Game of Diplomacy

János Kramár, Tom Eccles, Ian Gemp, Andrea Tacchetti, Kevin McKee, Mateusz Malinowski, Thore Graepel, Yoram Bachrach*

Supplementary Note 1 Diplomacy Game Rules Summary

Diplomacy [1] is a seven player board game revolving around negotiation and alliance formation in a way that accentuates tensions between cooperating and competing with others. The board consists of a map of Europe partitioned into provinces. Some of the provinces are **supply centers**, and allow the construction of **units**: armies, which move on the land, and fleets which move on the seas. Each player plays a **power** (country) and controls multiple units. Units capture supply centers by occupying the relevant province. To win the game, a player must gain control of the majority of the supply centers.

One unit may support another unit (owned by the same or another player), allowing it to overcome resistance by other units. Due to the inter-dependencies between units, players must coordinate the moves of their own units, and stand to gain by coordinating their moves with those of other players. Figure 1 depicts an example of interactions among several players, discussed below. We provide a brief overview of the core game mechanics. For a longer introduction, see earlier work on Diplomacy [2], and the rulebook [1].

Diplomacy features *simultaneous moves*: every turn each player writes down orders for *all* their units, without knowing what other players will do. Players then reveal their moves, which are executed simultaneously. The next position is fully determined by the moves and game rules, with no chance element (such as randomness originating in dice rolls).

Only one unit can occupy a province, and all units have equal strength. A unit may *Hold* (guard its province) or *Move* to an adjacent province. A unit may also *Support* an adjacent unit to hold or move, in order to overcome opposition by enemy units. Fleets, which move on the seas, may also *Convoy* armies (which otherwise only move on land), allowing them to move across multiple provinces in a single turn.

*Corresponding author, email: yorambac@google.com

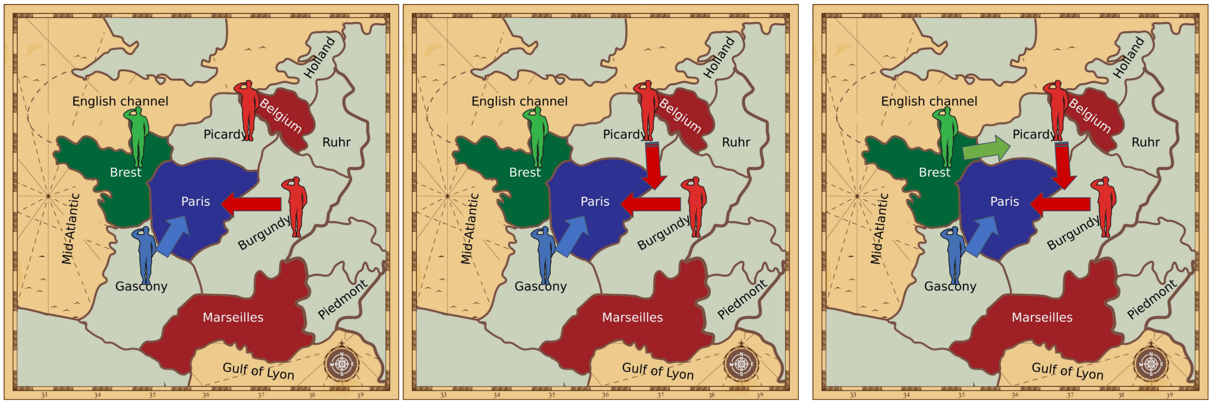

Supplementary Figure 1: Example of interactions between several players' moves. Left: initial moves with a standoff. Middle: a unit supporting another unit of the same player, resulting in the move succeeding. Right: cutting the support, resulting in a standoff. Background image by rawpixel.com and kjpargeter on Freepik.

In the example in Figure 1 we have three players: Red with units in Burgundy and Picardy, Blue with a unit in Gascony and Green with a unit in Brest. In the left of Figure 1, Red orders the unit in Burgundy to Move to Paris and simultaneously Blue orders the unit in Gascony to Move to Paris (with the remaining units holding). Units have equal strength so both sides fail and remain where they are. In the middle we consider a similar case, but where Red orders the unit in Picardy to Support the adjacent unit in Burgundy to enter Paris (making it two Red units against one Blue unit) so the Move succeeds. The right shows yet another similar scenario, where Blue asks for assistance from Green to avoid losing Paris; the unit in Brest Moves into Picardy, so the unit in Picardy must defend itself and can no longer Support the Move from Burgundy to Paris, so again we have a standoff and none of the units move.

The above example highlights several challenges in Diplomacy. The simultaneous resolution of moves means players must constantly reason about the actions of others. Further, cooperation between players is critical to success: Red may coordinate the moves of their own units in order to overcome resistance from Blue and enter Paris, however Blue may obtain the support of the Green player to overcome this. The design of Diplomacy is aimed at making sure that players must constantly form alliances and make agreements with others in order to achieve their goals.

In our Results section, we considered the Mutual-Proposal protocol, and our empirical analysis of this protocol was based on *Peace Contracts*. We now elaborate on such contracts building on the more detailed game description above. The goal of Peace contracts is to assure neither side attacks the other or supports a third party in doing so. Moving into a province held by a unit of the other side is considered a violation of the peace, and similarly, moving into a supply center province owned by the other side, or holding a supply center province owned by the other side. Finally, assisting a third party through a Support or

Convoy action is also considered a violation of the peace if the supported action would be considered a violation of the peace (i.e. assisting a third party in moving into a province held by the other side or a supply center owned by the other side, or in holding a supply center owned by the other side, is also considered an action violating the peace). A Peace Contract between p_i and p_j is a contract $D = (R_i, R_j)$ where R_i and R_j contain only the actions that do not violate the peace between p_i and p_j.

The heart of Diplomacy is negotiation between the players. Prior to the movement phase where players simultaneously move their units, there is a *negotiation phase*, where players communicate with each other in order to agree on what they might do in the next turns. People typically do this by talking to other players in private (or by using chat channels when playing online).

A key rule of Diplomacy is that the conversations during the negotiation phase are non-binding. No matter what a player promises to another player during the negotiation phase, they are free to choose any legal move they want during the movement phase (hence people sometimes refer to the negotiation phase as "cheap talk" [3]).

Supplementary Note 2 Hyperparameters Used in the Experiments

For reproducibility we provide the hyperparameter settings used in our experiments. In all negotiation algorithms, during the action phase we use $M = 4$ action profiles and $N = 128$ candidate actions, except for the learned-deviator experiments where we use $M = 2$ and $N = 64$. The experiments we run are quite computationally demanding, and increasing these values would have slowed experiments down (though likely higher values result in more accurate estimates and hence stronger agents).

For π^c we sample from the policy network with temperature 1, and for π^b we sample with temperature 0.1. For MBDS we use the same number of action profiles, $K = 8$ deal candidates, and $R = 10$ bargain rounds, with partner value weight of $\beta = \frac{1}{2}$ and bargain damping factor $\kappa = \frac{1}{2}$. For RSS we use an increased number of action profiles $M = 16$.

In addition, for our Sanctioning agents we use a sanctioning strength of $\alpha = 1$, finding that this balances between reducing the Conditional Deviators' winrates when there are few deviators and reducing the Sanctioning agents' effectiveness when there are many deviators: see Supplementary Note 10 for more details.

Supplementary Note 3 Limitations Of Our Approach

Diplomacy is an abstract game of strategy (similarly to Checkers, Chess or Go). Our methods are designed for large action spaces, in Diplomacy and other temporally extended environments where players take simultaneous actions. This might find applications in video

games and negotiation in economic settings or logistics domains where the scenario can be simulated sufficiently accurately. However, we assume perfect information apart from the negotiations, and a known model of deterministic environment transitions between turns. Most real-world applications are difficult to capture realistically using a simulator with a similar level of abstraction. Thus, significant further work is needed to apply such technology in real-world domains and to handle uncertainty in a robust way (in particular in relation to agreements that have been made.)

Further, we used limited protocols: they deal with a single timestep in the future, and do not cover counter-offers and re-negotiation. One reason for this was to narrow the search space to limit the computation budget. More work is required to cover more complex settings. Similarly, to keep our algorithms computationally feasible, they make some simplifying assumptions; a common one is assuming that other agents simply select actions from the unrestricted policy (rather than adhering to a restricted policy due to other contracts). Reasoning about agreements made by others significantly increases the computational complexity of our approaches, so we do so sparingly (for instance in the BATNA computation in MBDS). The Learned Deviator's performance might be limited by our choice of algorithm, such as the contracts they propose, or the features used to decide when to deviate. Hence other types of Deviators may require additional mechanisms for deterring deviations.

Additional limitations relate to the policy and value functions used, which are shared between all of the agents; this makes it easier for agents to agree on contracts, but is not common in real negotiation domains. Future work might drop this assumption and adopt the framework of Zero-Shot Coordination [4]. Furthermore, our methods hold the policy and value functions fixed following the initial reinforcement learning step. As the value function is trained through self-play of agents who do not communicate, it may not offer a good approximation of the true strength of board positions. It would be interesting to see whether incorporating additional reinforcement learning training could improve agent performance.

In this paper we study communication under a specific formal protocol rather than natural language. This has enabled us to better study the issue of deviations from agreements, because the meanings of agreements are unambiguous, making it clear when an agent carries an action that violates its past commitment. On the other hand, humans may find the protocols used in this work rigid or difficult to work with. Building agents who use natural language to negotiate in Diplomacy or other similar settings is clearly a very interesting area for future work. Studying non-binding negotiation in natural language, where agreements might be more fuzzy or subjective, would require additional datasets and substantial work. Publishing such research also requires first finding ways to mitigate the risks associated with natural language agents that may mislead others [5].

Finally, the focus in this work was on communication as a tool that enables agents to coordinate their actions by negotiating and agreeing on a joint plan for future moves. Communication can take on many other roles, such as conveying information or beliefs (about the state of the board or the goals or intents of other players), asking for information or

making demands. We only look at a specific use of communication, but show that even when only examining this restricted role, communication can dramatically improve agent performance in settings where cooperation is essential.

Supplementary Note 4 Convergence of the MBDS Approximation to the Nash Bargaining Solution

The negotiation algorithm we propose for the Propose-Choose protocol is Mutually Beneficial Deal Sampling (MBDS), aimed at approximating the Nash Bargaining Solution (NBS). The NBS is defined as selecting the deal that maximizes the product of utilities over the no-deal baseline (BATNA): $d^* = \arg\max_{(d_i,d_j)\in S}(d_i - d_i^*)(d_j - d_j^*)$, where $S \subset \mathbf{R}^2$ is the set of achievable expected values (d_i, d_j) for the agents under possible contracts d, and (d_i^*, d_j^*) is the value of the no-deal baseline for p_i and p_j respectively. For the purposes of this section, we assume that players p_i, p_j follow the agreed contract d, that the remaining players each follow their unrestricted policies π^b, and that bargaining does not take place (i.e. $R = 0$).

We make repeated use of Hoeffding's inequality [6], which bounds the probability that the sample average would exhibit a large deviation from its expected value:

Theorem 1 (Special case of Hoeffding's inequality)**.** *Let $X_1, \ldots, X_M$ be i.i.d. random variables taking values in $[-1, 1]$ with expectation $\mathrm{E}(X)$ and sample mean $\bar{X} = \frac{1}{M}\sum_{i=m}^{M} X_m$. Then $\Pr(\bar{X} - \mathrm{E}(X) > \delta) \leq \exp(-M\delta^2/2)$, and $\Pr(|\bar{X} - \mathrm{E}(X)| > \delta) \leq 2\exp(-M\delta^2/2)$.*

MBDS takes three hyperparameters: N, K, and M. MBDS uses a set of M action profiles $\mathbf{b}^1, \ldots, \mathbf{b}^M$ sampled for all of the players from their unrestricted policies π^b; it then constructs a set $\mathcal{D}_{i,j}$ of potential contracts by additionally sampling N candidate actions for each player, and selecting the sets of top K actions $C_{i,j} \subset A_i$ and $C_{j,i} \subset A_j$ according to a heuristic criterion (see MBDS algorithm) based on the estimated utility of those actions to players i and j, and then taking the Cartesian product $\mathcal{D}_{i,j} = C_{i,j} \times C_{j,i}$ of these action sets (hence considering a sample of K^2 possible contracts). For each candidate contract, MBDS performs M simulations of how the game might unfold under the contract. Each simulation is carried by taking the actions of p_i, p_j from the contract and taking actions for the remaining players from one of the action profiles $\mathbf{b}^1, \ldots, \mathbf{b}^M$.

We now prove that MBDS converges almost surely to the NBS top-ranked contract, i.e. the probability of selecting that contract tends to one, as N, K, M tend to infinity, if M tends to infinity faster than $\log N$ and $\log K$.

We begin by considering the no-deal baseline actions c_i^*, c_j^*. Their joint effect plays a crucial role in NBS and MBDS: not only does it show up in MBDS' heuristic for filtering candidate actions for contracts, but also in the formula NBS and MBDS use for ranking contracts. What's more, there's no guarantee that no-deal actions c_i^*, c_j^* chosen near-optimally based on sampling

produce a next-step game state close (in the value functions V_i and V_j) to that produced by the no-deal optimal actions $a_i^* = \text{argmax}_{a \in A_i} Q_i^{\pi_{-i}^b}(s,a)$, $a_j^* = \text{argmax}_{a \in A_j} Q_j^{\pi_{-j}^b}(s,a)$. The following lemma shows that if we choose high enough parameters N, M (with M growing faster than $\log N$), MBDS selects the correct no-deal baseline action a_i^* with high probability.

Lemma 1. *Let $a_i^{(1)}, \dots, a_i^{(N)} \in A_i$ be, in decreasing order, the N actions that produce the highest values of $Q_i^{\pi_{-i}^b}(s,a)$; for example, $a_i^{(1)} = a_i^*$. Let $\delta_i^{(n)} = Q_i^{\pi_{-i}^b}(s, a_i^{(1)}) - Q_i^{\pi_{-i}^b}(s, a_i^{(n)})$ be the performance gap between $a_i^{(n)}$ and $a_i^{(1)}$. Assume $\delta_i^{(2)} > 0$, so that the NBS solution is well-defined. Then*

$$\begin{aligned}\Pr(c_i^* = a_i^*) &\geq (1 - (1 - \pi_i^c(a_i^*))^N)\left(1 - \sum_{n=2}^{N} \exp(-M(\delta_i^{(n)})^2/2)\right) \\ &\geq (1 - (1 - \pi_i^c(a_i^*))^N)(1 - (N-1)\exp(-M(\delta_i^{(2)})^2/2)),\end{aligned}$$

so $Pr(c_i^* = a_i^*) \to 1$ as $N \to \infty$ and $M - 2\log N/(\delta_i^{(2)})^2 \to \infty$.

Proof. The probability of sampling a_i^* among the sampled candidates $c_i^1, \dots, c_i^N$ is $(1 - (1 - \pi_i^c(a_i^*))^N)$. Supposing it has been sampled, let n^* be the index such that $c_i^{n^*} = a_i^*$.

Now consider how c_i^* is selected using SAVE: for each action profile $\mathbf{b} \sim \pi_{-i}^b$, we compute $(V_i(T(s,(c_i^1,\mathbf{b}))), \dots, V_i(T(s,(c_i^N,\mathbf{b}))))$, an independent sample from an N-dimensional distribution. We use our M such samples $\mathbf{b}^1, \dots, \mathbf{b}^M$ to compute an N-dimensional sample mean, and then select c_i^* according to the index of the highest entry in that sample mean. For actions that were sampled *multiple* times, we can ignore all but one sample, since their respective $V_i(T(s,(c_i,\mathbf{b})))$ values will be equal.

For any $c_i^n \neq a_i^*$, the random variable $V_i(T(s,(c_i^{n^*},\mathbf{b}))) - V_i(T(s,(c_i^n,\mathbf{b})))$ is in the range $[-1,1]$, and its expectation is the performance gap $\delta^n = Q_i^{\pi_{-i}^b}(s, c_i^{n^*}) - Q_i^{\pi_{-i}^b}(s, c_i^n)$ between a_i^* and c_i^n. Theorem 1 then tells us that its sample mean $\hat{Q}_i^{B_{-i}}(s, c_i^{n^*}) - \hat{Q}_i^{B_{-i}}(s, c_i^n)$ will only fall below its expected value δ^n by more than δ^n with a probability that's at most $\exp(-M(\delta^n)^2/2)$. If the algorithm chooses $c_i^* = c_i^n$, then this sample mean must be negative; so this has probability at most $\exp(-M(\delta^n)^2/2)$. Aggregating using a union bound across all $c_i^n \neq a_i^*$, and again bearing in mind that duplicate candidates can be ignored, it follows that the chance of selecting an action $c_i^* \neq a_i^*$ is at most $\sum_{n=2}^{N} \exp(-M(\delta_i^{(n)})^2/2) \leq (N-1)\exp(-M(\delta_i^{(2)})^2/2)$.

Finally, because π_i^c has full support, we have $\pi_i^c(a_i^*) > 0$; together with the assumption that $\delta_i^{(2)} > 0$, this implies that $(1 - (1 - \pi_i^c(a_i^*))^N)(1 - (N-1)\exp(-M(\delta_i^{(2)})^2/2)) \to 1$ as $N \to \infty$ and $M - 2\log N/(\delta_i^{(2)})^2 \to \infty$. □

Having established that $c_i^* = a_i^*$ and $c_j^* = a_j^*$ with high probability for suitably large N, M, we now turn our attention to the candidate contract set (which we'll denote as $\mathcal{D}_{i,j}$). We aim to show that the NBS contract is one of the contracts in the set $\mathcal{D}_{i,j}$ that MBDS

considers. We show that as $K, N \to \infty$, *any* contract (and in particular the NBS contract), does indeed occur in $\mathcal{D}_{i,j}$. Recall that $\mathcal{D}_{i,j}$ is constructed by taking the Cartesian product of the top K actions $C_{i,j}$ and $C_{j,i}$ among $c_i^1, \ldots, c_i^N$ and among $c_j^1, \ldots, c_j^N$, according to a heuristic $\hat{Q}_i^{\mathbf{B}_{-i}}(s, c_i) + \beta \frac{q_{i,j}^0}{q_{j,i}^0} \hat{Q}_j^{\mathbf{B}_{-i}}(s, c_i)$ (and similarly for c_j).

We'll denote the heuristic evaluated using $c_i^* = a_i^*$ and $c_j^* = a_j^*$, and true expected values rather than sampling, as $h_i(c_i) = Q_i^{\pi_{-i}^b}(s, a_i) + \beta \frac{d_i^*}{d_j^*} Q_j^{\pi_{-i}^b}(s, a_i)$ (and similarly $h_j(c_j)$), and let $h_i^{(1)}, h_i^{(2)}, \ldots$ be the highest possible values of $h_i(a_i)$ among all actions $a_i \in A_i$.

Lemma 2. *Consider an action a_i that's among the top K actions according to h_i, i.e. such that $h_i(a_i) \geq h_i^{(K)}$. Then,*

$$\begin{aligned}
&\Pr(a_i \notin C_{i,j} \text{ and } (c_i^*, c_j^*) = (a_i^*, a_j^*)) \\
&\leq (1 - \pi_i^c(a_i))^N + \sum_{k=K+1}^{N} \exp(-M(h_i(a_i) - h_i^{(k)})^2 / (1 + \beta \tfrac{d_i^*}{d_j^*})^2 / 2) \\
&\leq (1 - \pi_i^c(a_i))^N + (N - K) \exp(-M(h_i(a_i) - h_i^{(K+1)})^2 / (1 + \beta \tfrac{d_i^*}{d_j^*})^2 / 2),
\end{aligned}$$

so $\Pr[a_i \notin C_{i,j}$ *and* $(c_i^*, c_j^*) = (a_i^*, a_j^*)] \to 0$ *as* $N \to \infty$ *and* $M - 2\log(N)(1 + \beta \frac{d_i^*}{d_j^*})^2 / (h_i(a_i) - h_i^{(K+1)})^2 \to \infty$.

Proof. The probability of sampling a_i among N independent samples from π_i^c is $1 - (1 - \pi_i^c(a_i))^N$, which approaches 1 as $N \to \infty$. If it is sampled and $(c_i^*, c_j^*) = (a_i^*, a_j^*)$ then the only way $a_i \notin C_{i,j}$ is if MBDS estimates a higher heuristic value for some a_i' with $h_i(a_i') \leq h_i^{(K+1)}$.

Let $h_i^m(a_i) = V_i(T(s, (a_i, \mathbf{b}_m^{-i}))) + \beta \frac{d_i^*}{d_j^*} V_j(T(s, (a_i, \mathbf{b}_m^{-i})))$ and likewise $h_i^m(a_i')$, and consider the random variables $X_m = (h_i^m(a_i') - h_i^m(a_i)) / (1 + \beta \frac{d_i^*}{d_j^*})$, which are i.i.d. with values in $[-1, 1]$, expected value $(h_i(a_i') - h_i(a_i)) / (1 + \beta \frac{d_i^*}{d_j^*})$, and sample mean that's just $1/(1 + \beta \frac{d_i^*}{d_j^*})$ times MBDS's estimate of the advantage of a' over a if $(c_i^*, c_j^*) = (a_i^*, a_j^*)$.

Applying Theorem 1, we conclude that the probability of the estimate being non-negative while $(c_i^*, c_j^*) = (a_i^*, a_j^*)$ is at most $\exp(-M(h_i(a_i') - h_i(a_i))^2 / (1 + \beta \frac{d_i^*}{d_j^*})^2 / 2)$; aggregating over different such a_i', we find the probability is bounded from above by $\sum_{k=K+1}^{N} \exp(-M(h_i(a_i) - h_i^{(k)})^2 / (1 + \beta \frac{d_i^*}{d_j^*})^2 / 2)$.

□

Corollary 1. *For any contract d and for sufficiently large K,* $\Pr[d \in \mathcal{D}_{i,j}$ *or* $(c_i^*, c_j^*) \neq (a_i^*, a_j^*)] \to 1$ *as $N \to \infty$ and $M / \log(N) \to \infty$. In particular, this is true for the NBS-optimal contract.*

MBDS selects the set $\mathcal{D}_{i,j}$ of contracts it considers through a procedure that samples actions from the agent policies. The results above show that $\mathcal{D}_{i,j}$ indeed contains the NBS-optimal contract with high probability when the parameters K, N are high enough. Rather

than applying this sampling procedure, we could instead simplify MBDS while ensuring the NBS-optimal contract is considered, by employing an exhaustive search through all possible contracts. However, the vast majority of possible actions a player can take are poor both for working in isolation and for coordinating with a peer and obtaining their help. By sampling actions from the policy, we focus the contract actions on ones that are likely to yield good outcomes. Further, the heuristic selection method for candidate contracts helps with efficiently finding actions that help both players, without incurring the substantial computational cost of evaluating $M \cdot N^2$ game states, one for every action profile (for the other players) and pair of candidate actions.

Nonetheless, if N and K are sufficiently large, then the sampling procedure will sample each and every one of the finite number of possible actions, so all of them will occur in the top K actions, and as a result $\mathcal{D}_{i,j}$ will contain every possible contract. According to the final appendix of [7], there are game states in Diplomacy where the joint action space among 7 players had size $10^{64.3}$. Therefore, there must be a player with action space of size at least $\sqrt[7]{(10^{64.3})} = 10^{9.2}$; so to sample all contracts, K must be at least $10^{9.2}$. Further, even in the best case, in which π^c is uniform, the expected waiting time before sampling all actions would be $\approx 10^{9.2} \cdot \log(10^{9.2}) = 10^{10.5}$; so N must be at least $10^{10.5}$ to achieve high probability of sampling all actions at least once.

Having shown that for large K, N the set $\mathcal{D}_{i,j}$ of contracts considered by MBDS includes the NBS-optimal contract with high probability, we turn to showing that by increasing the parameter M we can ensure that with high probability, MBDS finds a top-performing contract according to the NBS criterion. The parameter M controls the number of simulations we perform to estimate the player utilities for each contract. Intuitively, as this number increases, our estimates get closer to the true expected player utilities, and as a result we rank the contracts in the correct order and select the true NBS contract.

Now, we define a *δ-suboptimal contract* as being a contract whose NBS score is lower than that of the best contract in $\mathcal{D}_{i,j}$, by more than δ. We show that for any $\delta \geq 0$ the probability that MBDS selects a δ-suboptimal contract can be made negligibly low by taking the parameter M to be high enough, so long as we've also taken M, N high enough to ensure that the right no-deal baseline actions are selected with high probability.

Lemma 3. *Consider the best contract $d \in \mathcal{D}_{i,j}$ according to the NBS criterion. For any $\delta > 0$, the probability that $(c_i^*, c_j^*) = (a_i^*, a_j^*)$ and yet MBDS selects a δ-suboptimal contract, is at most $6(K^2-1)\exp(-M\delta^2/32) + \exp(-M(d_i - d_i^*)^2/2) + \exp(-M(d_j - d_j^*)^2/2)$. Hence, this probability tends to zero as $M \to \infty$.*

Proof. Let $d = (\{a_i\}, \{a_j\})$, and consider a δ-suboptimal contract $d' = (\{a'_i\}, \{a'_j\}) \in \mathcal{D}_{i,j}$ i.e. a contract such that $\max(0, d_i - d_i^*) \cdot \max(0, d_j - d_j^*) - \max(0, d'_i - d_i^*) \cdot \max(0, d'_j - d_j^*) > \delta$. Note that if d's NBS score is zero then no such d' exists, so the conclusion of the lemma trivially holds; so let's assume d's NBS score is positive.

Now define $\Delta_{i,m} = V_i(T(s, (a_i, a_j, \mathbf{b}^m_{-\{i,j\}}))) - V_i(T(s, (a_i^*, a_j^*, \mathbf{b}^m_{-\{i,j\}})))$, and analogously $\Delta_{j,m}, \Delta'_{i,m}, \Delta'_{j,m}$. Note that these are random variables with expected values $d_i - d_i^*, d_j -$

$d_j^*, d_i' - d_i^*, d_j' - d_j^*$ respectively. We denote their sample means as $\bar{\Delta}_i, \bar{\Delta}_j, \bar{\Delta}_i', \bar{\Delta}_j'$.

We now apply Theorem 1 to $\bar{\Delta}_i$ and $\bar{\Delta}_j$, which take values in $[-1, 1]$, and obtain that $\Pr(\bar{\Delta}_i < 0 \text{ or } \bar{\Delta}_j < 0) \leq \exp(-M(d_i - d_i^*)^2/2) + \exp(-M(d_j - d_j^*)^2/2)$.

MBDS chooses d' over d if it estimates that $\max(0, \hat{d}_i' - \hat{d}_i^*) \cdot \max(0, \hat{d}_j' - \hat{d}_j^*) > \max(0, \hat{d}_i - \hat{d}_i^*) \cdot \max(0, \hat{d}_j - \hat{d}_j^*)$, where $\hat{d}_i = \hat{Q}_i^{\mathbf{B}_{-\{i,j\}}}(s, (a_i, a_j))$, $\hat{d}_i^* = Q_i^{\mathbf{B}_{-\{i,j\}}}(s, (c_i^*, c_j^*))$, and analogously with $\hat{d}_i', \hat{d}_j, \hat{d}_j^*, \hat{d}_j'$. Note that if $c_i^* = a_i^*$ and $c_j^* = a_j^*$ then $\bar{\Delta}_i = \hat{d}_i - \hat{d}_i^*$ (and analogously for $\bar{\Delta}_j, \bar{\Delta}_i', \bar{\Delta}_j'$); if additionally $\bar{\Delta}_i, \bar{\Delta}_j \geq 0$ then MBDS's estimated advantage of d' over d will be:

$$
\begin{aligned}
&\max(0, \bar{\Delta}_i') \cdot \max(0, \bar{\Delta}_j') - \bar{\Delta}_i \bar{\Delta}_j \\
&\leq \max(0, \bar{\Delta}_i' \bar{\Delta}_j') - \bar{\Delta}_i \bar{\Delta}_j \\
&\leq \max(0, \bar{\Delta}_i' \bar{\Delta}_j' - \bar{\Delta}_i \bar{\Delta}_j) \\
&\leq \max(0, \bar{\Delta}_i' \bar{\Delta}_j' - \bar{\Delta}_i \bar{\Delta}_j + (\mathrm{E}(\Delta_i)\,\mathrm{E}(\Delta_j) - \mathrm{E}(\Delta_i')\,\mathrm{E}(\Delta_j')) - \delta) \\
&= \max(0, -\delta + \bar{\Delta}_j'(\bar{\Delta}_i' - \bar{\Delta}_i) - \mathrm{E}(\Delta_j')\,\mathrm{E}(\Delta_i' - \Delta_i) + \bar{\Delta}_i(\bar{\Delta}_j' - \bar{\Delta}_j) - \mathrm{E}(\Delta_i)\,\mathrm{E}(\Delta_j' - \Delta_j)) \\
&= \max(0, -\delta + \bar{\Delta}_j'(\bar{\Delta}_i' - \bar{\Delta}_i - \mathrm{E}(\Delta_i' - \Delta_i)) + (\bar{\Delta}_j' - \mathrm{E}(\Delta_j'))\,\mathrm{E}(\Delta_i' - \Delta_i) \\
&\qquad + \bar{\Delta}_i(\bar{\Delta}_j' - \bar{\Delta}_j - \mathrm{E}(\Delta_j' - \Delta_j)) + (\bar{\Delta}_i - \mathrm{E}(\Delta_i))\,\mathrm{E}(\Delta_j' - \Delta_j)).
\end{aligned}
$$

Looking at the terms in the sum aside from $-\delta$, we can apply Theorem 1 to $\bar{\Delta}_j', \bar{\Delta}_i$, obtaining probability $\leq \exp(-M\delta^2/32)$ that the second and fourth term are $> \delta/4$, and to $\bar{\Delta}_i' - \bar{\Delta}_i, \bar{\Delta}_i' - \bar{\Delta}_j$, obtaining probability $\leq 2\exp(-M\delta^2/32)$ that the first and third term have absolute value $> \delta/4$. As a result, the estimated advantage can only be non-negative with probability at most $6\exp(-M\delta^2/32)$. Aggregating this (using a summation bound) over all $K^2 - 1$ alternate contracts $d' \in \mathcal{D}_{i,j} \setminus \{d\}$, and combining with the probability that $\bar{\Delta}_i, \bar{\Delta}_j \geq 0$, the result follows.

□

There are three ways MBDS could fail to select a contract with a high Nash Bargaining Score. First, it could select the wrong no-deal baseline action; second, it could fail to include any strong candidates in the deal candidate set $\mathcal{D}_{i,j}$; and third, it could fail to select a strong contract even if there is one in the candidate set. These are respectively addressed by Lemmas 1-3, which provide bounds on the probabilities of these failure modes. Combining them, we arrive at our main theorem.

Theorem 2. *Consider a contract $d = (\{a_i\}, \{a_j\})$ with positive NBS score, and where a_i, a_j are among the top K actions in A_i, A_j respectively according to h_i and h_j. Then the probability that MBDS selects a contract with Nash Bargaining Score more than δ worse than that of d*

is at most:

$$
\begin{aligned}
&(1-\pi_i^c(a_i^*))^N + (1-\pi_j^c(a_j^*))^N + (1-\pi_i^c(a_i))^N + (1-\pi_j^c(a_j))^N \\
&+ (N-1)\exp(-M(\delta_i^{(2)})^2/2) + (N-1)\exp(-M(\delta_j^{(2)})^2/2) \\
&+ (N-K)\exp(-M(h_i(a_i) - h_i^{(K+1)})^2/(1+\beta\tfrac{d_i^*}{d_j^*})^2/2) \\
&+ (N-K)\exp(-M(h_j(a_j) - h_j^{(K+1)})^2/(1+\beta\tfrac{d_j^*}{d_i^*})^2/2) \\
&+ \exp(-M(d_i-d_i^*)^2/2) + \exp(-M(d_j-d_j^*)^2/2) + 6(K^2-1)\exp(-M\delta^2/32),
\end{aligned}
$$

We note that the above expression tends to 0 *as* $N \to \infty$ *and* $M/\log(N) \to \infty$*.*

Corollary 2. *Supposing there is a single NBS-optimal contract, if we set* K *high enough then the NBS-optimal contract is chosen with probability that tends to* 1 *as* $N \to \infty$ *and* $M/\log(N) \to \infty$*. We also obtain the same result if* $N \to \infty$*,* $K \to \infty$*,* $M/\log(\max(N, K)) \to \infty$*.*

Regarding the applicability of this theorem, it's worth bearing in mind that neither NBS nor MBDS makes allowances for not knowing the true value function; for our algorithms we simply assume the value function V_i we get from our neural network is good enough.

Supplementary Note 5 Comparison and Extensions of our Diplomacy Negotiation Methods

We have proposed two protocols for making agreements regarding actions to be taken in the next step: the Mutual Proposal protocol and the Propose-Choose protocol. We have also proposed the RSS method for making agreements under the Mutual Proposal protocol, and the MBDS method for the Propose-Choose protocol. We now compare these protocols, and consider how they could be extended.

Both the Mutual Proposal and Propose-Choose protocols achieve gains from negotiation. However, in certain regards, they lie on opposite ends of a spectrum relating to the expressiveness of the language of contracts. The Propose-Choose protocol allows for a very large number of possible contracts, each of which is maximally restrictive regarding the next actions. This means that under this protocol it is difficult to select the best contract and agree on it. However, on the flip side, this also means that under this protocol it is easy to see what might occur if a contract is agreed and followed, and easy to find a contract to take advantage of any given mutually beneficial opportunities on the board.

On the other hand, the Mutual Proposal protocol allows for exactly one possible contract (given the game state) between any pair of players. This makes it trivially easy to decide which contract to propose, but at the same time this means that it is harder to predict what might happen if a contract is agreed, since the contract only rules out a subset of the actions.

Further, this protocol crucially relies on the *contract type*, i.e. the actions are are disallowed when a contract is agreed between two powers. We use the term "contract type" to refer to the rule that takes a board state and specifies a single contract, i.e. a restricted set of permitted actions to be taken in the next time step.

For example, we used the Peace contract type, whose meaning is that neither partner may perform a Move to a province containing the other partner's unit or supply center, or provide a Support or Convoy for another unit to do so. This depends on the game state: for example, the Peace contract disallows moving a unit into a province when the other side has a unit in that province, but if the province is empty and isn't a supply center, the Peace contract does allow moving a unit into that province. It's common in Diplomacy that two neighboring powers allocate some of their units to holding each other off, thus leaving them with fewer available units for other operations — in this case, a Peace contract could allow them to make better use of their resources.

Other contract types for the Mutual-Proposal protocol are possible, and the same RSS algorithm, as is, can be used with these contract types. Some examples include No-Move contracts forbidding armies from moving to *any* adjacent province, or No-Convoy contracts forbidding fleets from convoying units, or No-Support contracts that disallow either of the partners to support the units of any third party in their moves. These contracts impose restrictions that are less selectively targeted toward the actions the partners take to defend against each other, so they're less likely to be of mutual benefit. Each such contract type incorporates a different rule to determine which actions are allowed in a given state. When RSS simulates how the game might unfold, the contract type determines which actions may not be taken under the contract, but the RSS algorithm itself is unchanged.

There may also be refinements on the Peace contract type that produce greater benefit. For example a more sophisticated contract type might refer to the no-deal baseline actions, identify all sets of units whose actions are in direct opposition, and agree to "stand down" those units from opposing the other partner's actions. This would come at the cost of ambiguity about whether the contract has been broken, since there is no common knowledge about what the no-deal baseline actually is. One way to resolve this difficulty would be for a "reference player" to make common-knowledge predictions of what the players will do next.

There has been work on more elaborate negotiation protocols for Diplomacy, eg DAIDE [8], but it was typically done with an emphasis on being interoperable with human play, and less emphasis on well-defined semantics; so DAIDE has a concept of a Peace agreement as well, but the meaning is unspecified.

Exploring the spectrum between Mutual-Proposal and Propose-Choose further, another possibility would be to make Propose-Choose more realistic by agreeing on actions for only a subset of units (typically only a subset of units of each partner is of clear strategic interest to the other). The result would be a contract that's less restrictive than the all-units contracts we've used for Propose-Choose; this could also allow additional benefits by making agreements with more than one partner. However this would also require some RSS-like sampling to estimate the effects of a deal, and it may require a more elaborate algorithm to arrive at the

right subsets of units to make agreements about. We did not explore these further because the significant gains from the Mutual-Proposal and Propose-Choose protocols with the RSS and MBDS algorithms were enough to suit our purposes while being different enough to make a case that our conclusions weren't obtained solely due to incidental details of one method of negotiation.

Supplementary Note 6 Generalizing our Negotiation Methods Beyond Diplomacy

We have only tried applying the Mutual-Proposal and Propose-Choose protocols and the RSS and MBDS algorithms on the Diplomacy game. However, these methods could also be applied in other settings.

Our methods rely on simulating the next timestep, which requires a deterministic simulator of the environment. Games such as Diplomacy have deterministic rules, where the next timestep only depends on the current board state and the actions taken by the players. However, the algorithms may also be adapted to stochastic games, where the next timestep also depends on random variables, such as dice throws.

Our algorithms consider settings where actions are taken simultaneously, and where single-timestep agreements are meaningful enablers of coordination. Further, the methods require mutual knowledge of what every player's action space, policy, and value function are (which is needed for the SAVE and SVE algorithms to work).

Settings where our methods may be applicable include supply-chain or fleet-management domains. In such settings, multiple parties make simultaneous decisions regarding the procurement of items or selling of goods, or on how to move multiple vehicles in a fleet.

When parties are affected by the actions of other parties, agreements might allow the sides to better coordinate and jointly improve their utility. For instance, agreeing on selecting different routes for various vehicles may reduce congestion on a road by limiting the number of vehicles that use it.

A simulator for the above domains would take in the current state and player actions, and output the next state. For instance, in a fleet-management domain, the simulator could take in the current locations of vehicles in the fleet, and choices regarding the routes taken by vehicles (e.g. turning left at a certain intersection leads to a vehicle using one road instead of the other); the simulator could then determine the congestion level on each road, and the resulting travel times.

The Mutual-Proposal protocol requires a suitable mutually-beneficial contract type: for example, it could represent an agreement for vehicles carrying assorted goods to meet and exchange cargo to optimize their future itineraries. The Propose-Choose protocol uses a much larger space of contracts encompassing the combined action assigned to every unit; this could be applied to jointly optimizing vehicular route assignments.

Supplementary Note 7 Relation to Work on Computational Negotiation Mechanisms and the Evolution of Cooperation

Our work is closely related to a long-standing line of research in artificial intelligence examining computational negotiation mechanisms. Many approaches have been proposed for allowing agents to negotiate with one another across many environments. This is a very broad topic, as shown by various surveys that examine such methods and their limitations [9, 10, 11, 12, 13, 14, 15, 16, 17, 18, 19, 20]. The negotiation protocols we have proposed for Diplomacy are simple. We show that even such simple protocols give agents who use them a significant advantage over non-communicating agents. Further, having non-binding agreements under these protocols already gives rise to fascinating problems regarding deviation from agreements.

Our design of the Defensive agents is influenced by Axelrod's line of work on the Evolution of Cooperation [21], where retaliation strategies are designed such that the immediate gains from deviation are offset by losses in the future as a result of the Defensive agents changing their behavior in retribution to the deviation. This is sometimes referred to as the "Shadow of the Future" [22]. The setting studied in the Evolution of Cooperation work [21] and subsequent work is that of a repeated game, where participants play the same simple stage game such as Prisoner's Dilemma repeatedly with the same partner. More generally, repeated games, where multiple players play a simple stage game repeatedly with one another, were used to examine related topics such as mixing strategies to reduce exploitability, strategically withholding information, misrepresenting intents or bluffing [23, 24, 25, 26].

Real-world negotiation has a number of attributes that differ from repeated stage games, and that Diplomacy captures more closely. Negotiating actors may be sometimes in conflict regarding some issues and interests, and may sometimes, even at the same time, be cooperating on some other issues. Actors may have a number of different negotiating partners to choose from, each with their own differing strengths, goals, and plans. Further, rather than taking turns to respond to each other's moves, real-world negotiators are often managing multiple relationships in parallel.

More formally, our research complements work on repeated games and the Evolution of Cooperation as Diplomacy differs from repeated games and Iterated Prisoner's Dilemma in multiple ways. First, we consider a *single* game that is temporally extended, rather than a repeated stage game. Hence, in a single Diplomacy game agents are extremely unlikely to meet under the exact same circumstances, but rather interact again in different board states, in which their relative strength may be different. This means that the possible gains from deviation can vary widely across the game.

Furthermore, in our case the Shadow of the Future is the result of a change in the behavior of the Defensive agent in subsequent turns of a single game, rather than multiple games. For *infinitely repeated* stage games there are prominent results in game theory stating

that there exist fully cooperative Nash equilibria [27, 28], but as our setting relates to a single temporally extended game, such results do not apply (and indeed, establishing a fully cooperative equilibrium proves to be challenging).

For the setting of a single Diplomacy game, sanctioning deviation from contracts ameliorates the problem of strategic deviation from past agreements, but the Learned Deviator still outperforms full truthful Sanctioning peers. However, having players interact repeatedly in multiple Diplomacy games extends the interaction time horizon, resulting in a larger potential future loss upon a deviation, which could increase the incentive of agents not to break agreements. This could be a very interesting avenue for future research.

We further note that as Diplomacy has an incredibly large action space, agents may consider a very large space of behaviors. This is in contrast to only two strategies in Prisoner's Dilemma, "cooperating" or "defecting". In Diplomacy the undesired behavior, of agreeing to a contract and then selecting an action that violates the contract, might take on many forms, as there are many possible ways to deviate from an agreed contract.

Finally, the different modes of behavior change, ceasing to communicate for Binary Negotiators and active retaliation for Sanctioning agents, produce different effect sizes. Ceasing to communicate can be viewed as simply ceasing to trust that the other side would adhere to agreements, whereas sanctioning takes a more active role in lowering the deviator's utility. Iterated Prisoner's Dilemma cannot capture such different effects due to its very limited strategy space.

Supplementary Note 8 On the Computational Difficulty of Solving the Game of Diplomacy

Diplomacy is interesting as it is an externally defined AI challenge and a game played by people (similarly to Chess, Backgammon or Stratego). Further, it has seven players and is thus richer than two-player zero-sum games, and can serve as a good example of a setting where players must form temporary alliances with some peers so as to overcome others. Another realistic feature of Diplomacy is the need to take multiple interrelated decisions simultaneously (in Diplomacy this relates to deciding how to move multiple units all at once). However, these exact properties that make Diplomacy interesting also pose a computational barrier for game theoretic analysis, which typically relies on solving games for a Nash equilibrium, or refinements such as a subgame perfect equilibrium [29].

Solving for a Nash equilibrium is computationally hard [30, 31], making this a challenge even in small games. As we discuss in the introduction, Diplomacy is an enormous game. Selecting moves for multiple units at the same time results in a vast action space of 10^{21} to 10^{64} legal actions per turn, and a rough estimate of the game tree size of Diplomacy is 10^{900} [7] (in the introduction we contrast this with the game of Chess, which has fewer than 100 legal actions per turn, and a game tree size of 10^{123}). The sheer size of the game tree of Diplomacy and the computational complexity of applying game theoretic analysis means

that in practice using such tools to build AI agents requires solving complex algorithmic challenges [32, 7, 33].

Similarly to recent work on smaller games such as Chess, Backgammon, or Go [34, 35, 36] we chose to use a reinforcement learning based method to identify strong agent policies. Our approach combines reinforcement learning with game theoretic principles such as the Nash Bargaining Solution [37, 38], applying a Monte-Carlo technique to overcome computational barriers. Such a combination of reinforcement learning and algorithmic game theory methods could fuel further successes in developing agents in the future.

Supplementary Note 9 Analysis of the Learned Deviator Behavior

We provide further analysis of the behavior patterns of the Learned Deviator, in its optimal parameter setting ($t_\phi = 0.15, t_\psi = 0.03$ for Mutual Proposal, or $t_\phi = 0.25, t_\psi = 0.03$ for Propose-Choose), when playing against a population of six Sanctioning agents.

As introduced in the section on the Learned Deviator, the parameter t_ϕ relates to minimum immediate improvement in value required to consider a deviation, and t_ψ relates to the maximum remaining opponent strength to allow a deviation. One way to understand the impact of different settings of t_ϕ and t_ψ is to look at how they affect the probability that at least one deviation will occur; we show this in Figure 2.

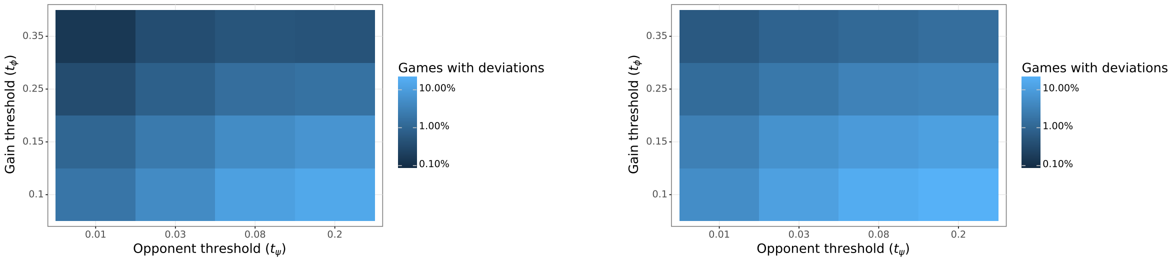

Supplementary Figure 2: Probability of any deviations occurring, in games with one Learned Deviator and six Sanctioning agents.

This figure, which reuses our sweep over the parameters t_ϕ, t_ψ (in the main text), indicates that the Learned Deviator performance tends to be more sensitive to changes in t_ψ than in t_ϕ. For instance, with Mutual-Proposal, under the optimal parameters $t_\phi = 0.15, t_\psi = 0.03$, the Deviator advantage $\frac{W_{dev}}{W_{san}}$ is 1.017. Fixing $t_\phi = 0.15$ and setting t_ψ to the larger values of 0.08, 0.2, 0.35 and 0.5, we get Deviator advantage of 1.012, 0.997, 0.991, 0.984 respectively. Under the strongest settings, the value of $t_\psi = 0.03$ means that the Learned Deviator is quite conservative and only deviates when the remaining opponent strength is quite low. As a result, in many games where a Learned Deviator plays against Sanctioning agents, no deviation occurs. (Similarly, with Propose-Choose, the optimum was at $t_\phi = 0.25, t_\psi = 0.03$,

producing Deviator advantage of 1.010; setting t_ψ to the larger values of 0.08, 0.2, 0.35, 0.5 produces Deviator advantage of 1.010, 1.001, 0.994, 0.987 respectively.)

Figure 3 shows the winners in games where a deviation occurred. It shows that while in the majority of games no deviation occurs, when a deviation does occur, the deviator is typically the winner; Further, in quite a few cases (particularly with Mutual-Proposal), the deviation target with whom a contract was broken was the winner. This suggests that Learned Deviators often make their first deviation against a strong opponent.

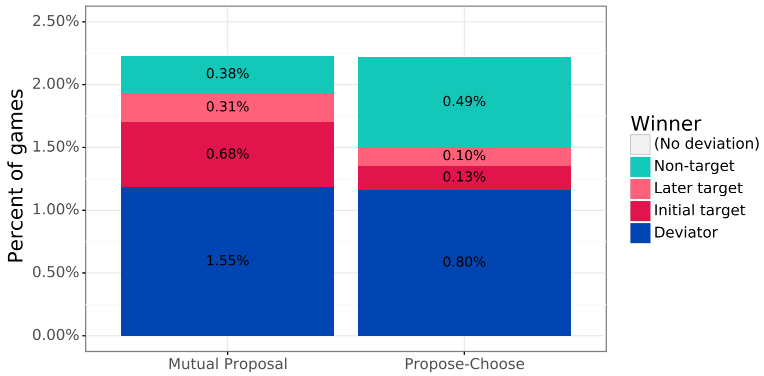

Supplementary Figure 3: Winners of games with one Learned Deviator and six Sanctioning agents, in which deviations occur. The percentages are relative to *all* games, the majority of which have no deviations occurring in them (i.e. the colored bars sum up to less than 3%, as deviations occur in less than 3% of the games)

Figure 4 shows a histogram of the number of turns until the first deviation, in games where a contract was broken. It shows that deviations rarely occur early in the game, meaning that the Learned Deviator honors its agreements until quite late in the game (often very late in the game), especially under the Mutual Proposal protocol. For comparison, the average number of contract-turns in the whole game for the deviator is 12.4 for the Mutual Proposal protocol and 9.5 for the Propose-Choose protocol – or if we consider only games where the deviator wins, 15.8 and 10.3, respectively.

To highlight the behavior of the Learned Deviator, we provide a similar histogram of the number of contract-turns until the first deviation, but this time considering the Conditional Deviator, shown in Figure 5. Figure 5 indicates that Conditional Deviators indeed deviate from contracts very early in the game. For instance, in the Propose-Choose protocol, in many cases the Conditional Deviator breaks the first contract it agrees to. Hence, when a Conditional Deviator plays against Sanctioning agents, deviations occur early in the game, leaving a long time horizon for the Sanctioning Agents to respond to the deviation and resulting in the low performance of the Conditional Deviator against the Sanctioning agents.

Figure 6 shows the number of peers with whom the Learned Deviator breaks contracts. Once a Sanctioning agent notices a deviation from a contract, it never agrees to another contract with the deviator, so the Learned Deviator may break at most a single contract

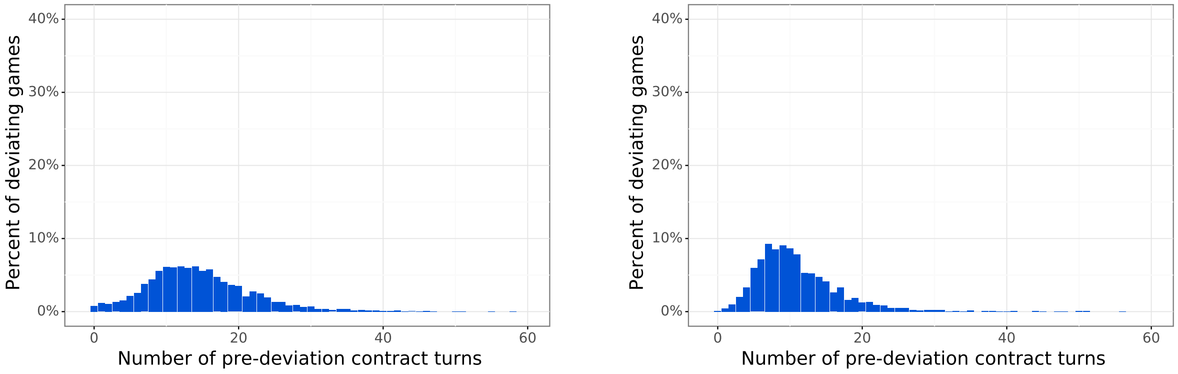

Supplementary Figure 4: Number of contract-turns before the first turn in which a contract is broken in games where the Learned Deviator indeed breaks a contract; cases are broken down by how many opponents were active at the time of first deviation. (Left: Mutual-Proposal, Right: Propose-Choose)

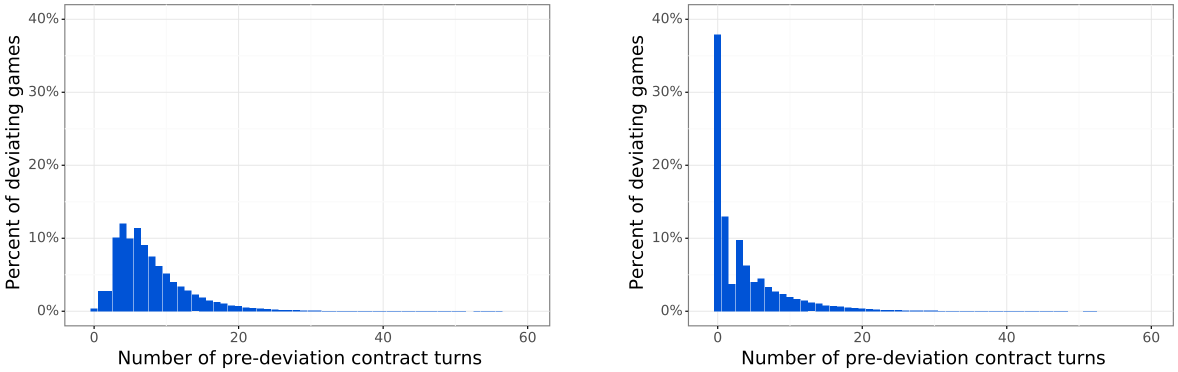

Supplementary Figure 5: Number of contract-turns before the first turn in which a contract is broken in games where a Conditional Deviator plays against Sanctioning Agents (Left: Mutual-Proposal, Right: Propose-Choose)

with each of the six players. However, as the figure shows, in the vast majority of games with deviations, the Learned Deviator only breaks contracts with one or two of its counterparts, even if more opponents remain active at the time of first deviation.

Supplementary Note 10 The Impact of the Sanctioning Parameter α

The behavior of our Sanctioning Agents depends on a parameter α. Such an agent ranks actions based on two values: its own value $V_i(s')$ and the deviator's value $V_j(s')$. The agent aims to maximize the metric $V_i(s') - \alpha V_j(s')$ with the parameter α determining the relative

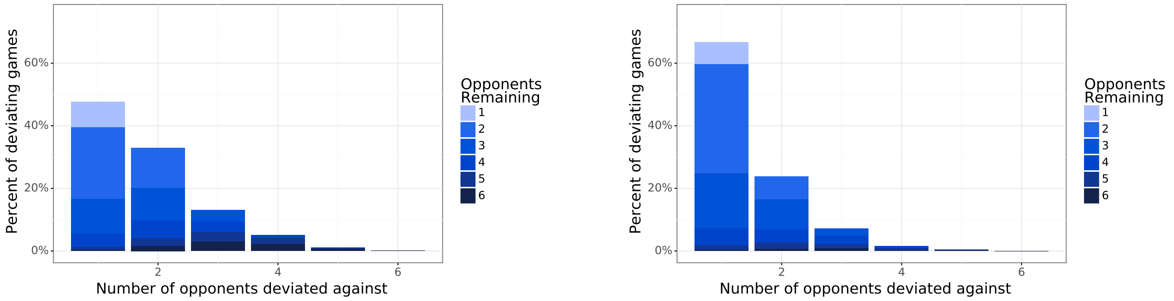

Supplementary Figure 6: Number of deviated-against opponents in games where the Learned Deviator indeed breaks a contract (Left: Mutual-Proposal, Right:Propose-Choose)

importance the agent places on its own probability $V_i(s')$ of winning, versus on lowering p_j's probability $V_j(s')$ of winning. For $\alpha = 0$ we recover the no-sanctioning behavior of Baseline Negotiators, while for $\alpha \to \infty$ the focus is only on making the deviator lose the game.

We consider games with k Conditional Deviators playing against $7 - k$ Sanctioning agents, showing in Figure 7 the effect that the parameter α has on the winrates of the agents. We see that when there is a single Conditional Deviator, higher values of α, i.e. more sanctioning behavior, results in lower winrates for the Deviator. In games where there are more Deviators, the relation is more complicated, and the figure also shows how sanctioning can be costly. Very high values of α mean that the Sanctioning agents focus on punishing a Deviator so much that they neglect their own position; as a result we can see that beyond a certain point, increasing α is no longer effective in further lowering the winrate of the Deviators. Unsurprisingly, a single Sanctioning Agent performs progressively worse against 6 Conditional Deviators as soon as $\alpha > 0$.

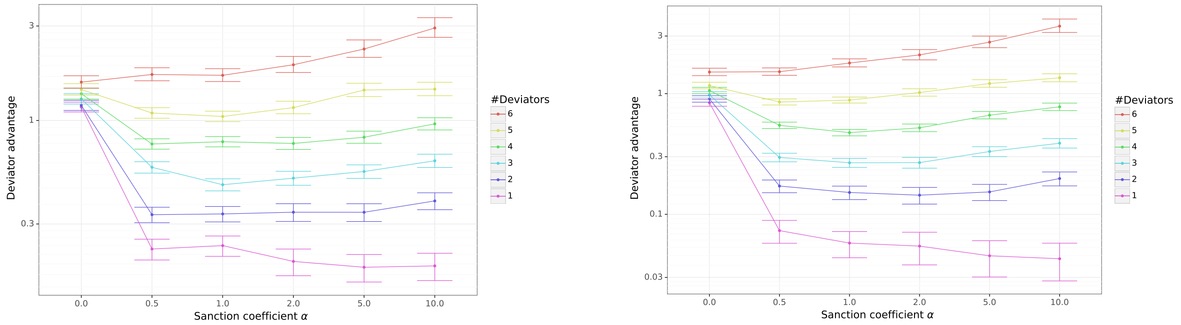

Supplementary Figure 7: Impact of the Sanctioning parameter α (Left: Mutual-Proposal, Right:Propose-Choose)

Supplementary Note 11 Stability Analysis of Agent Cooperative Behavior

Our discussion in the Results section considered various agent behaviors. We started with non-communicating agents and showed that they are outperformed by Baseline Negotiators. In turn, the Baseline Negotiators are outperformed by Simple and Conditional Deviators who may break their past agreements. To deter such deviations, we considered Defensive agents such as the Binary Negotiators and Sanctioning agents; for the Propose-Choose protocol both Binary Negotiators and Sanctioning agents outperform the Deviator agents in games where there are few Deviators, but for the Mutual-Proposal protocol only the Sanctioning agents outperform Deviators (though the Binary Negotiators do significantly reduce the advantage of Deviators as compared to Baseline Negotiators). While Sanctioning agents outperform Deviators, under both protocols, we showed that the more sophisticated Learned Deviator, whose behavior is optimized against a population of Sanctioning agents, indeed slightly outperforms these agents. While each agent type we considered achieved an advantage over some previous agent type, we note that this does not mean that each newly introduced agent outperforms *all* the agents introduced previously. For example, a Learned Deviator playing against many Conditional deviators would have a lower winrate.

We now consider whether communication and cooperative behavior are likely to persist in a population of learning agents. Our analysis is based on the theoretical foundation of the Nash equilibrium [39], one of the cornerstones of game theory [29]. We consider a set of strategies which are possible agent behaviors, consisting of the agents types discussed above. A *strategy profile* consists of a strategy (agent type) for each of the players in the game. Such a strategy profile is said to be at a Nash equilibrium if no participant can obtain a higher utility (winrate) by unilaterally switching to a different strategy. Given a strategy profile (i.e. the types of all the participating agents), we use a Monte-Carlo approach to evaluate the expected winrate for each of the agents: we run many games with agents of the specified types, and measure the proportion of the games won by agents of each type (we assign each agent to a random country in these games).

Since this approach only provides a noisy estimate of the agent winrate, we used the slightly relaxed concept of an ϵ-Nash equilibrium [40] (sometimes called a near-equilibrium). A strategy profile is at a near-equilibrium if no agent can gain more than ϵ by unilaterally switching their strategy; in our case, this means that a profile of agent types is at an ϵ-equilibrium if no agent can improve its winrate by more than ϵ by switching to a different agent type.

We first note that a set of agents who are *all* non-communicating agents is an equilibrium with respect to the use of communication. No agent has an incentive to unilaterally start using communication, since no other agent would respond to its communication. However, attempting to communicate also does not lead to a degradation in performance for that agent, and when a population of agents includes a non-zero fraction of communicators (Baseline

Negotiators), there is an incentive for other agents to start communicating. We now apply the Nash equilibrium concept to determine whether communication and cooperative behavior could persist in a population of learning agents.

Section 11.1 examines the stability of a population of Sanctioning agents, showing that this is a near-equilibrium (for ϵ=0.3%). Section 11.2 considers agents that mix the behavior of Learned Deviator and Sanctioning agents. These agents sanction other agents who break their agreements, but are also willing to themselves deviate from their own past agreements. We show that a population of such agents is another near-equilibrium (with ϵ=0.2%). Finally, in Section 11.3 we investigate whether these near-equilibria are similar to a full Nash equilibrium (an ϵ-equilibrium where $\epsilon = 0$), and more generally whether the defenses we've introduced allow for a true equilibrium with mostly-honest cooperation.

11.1 Near-Stability of Honest Communication

Consider a population of $n = 7$ Sanctioning agents, and one of these agents that could potentially switch its behavior to that of a Deviator. Each Sanctioning agent is playing against other Sanctioning agents that never break contracts, and thus never triggers their retaliation behavior, giving each such agent the full gains from communication. On the other hand, our results show that switching to being a Simple or Conditional Deviator results in the agent significantly reducing its winrate.

In our analysis of the Learned Deviator, the majority of the possible parameter thresholds t_ϕ, t_ψ considered as potential settings for a Learned Deviator result in a decrease in winrate (as compared to being a Sanctioning agent which does not deviate from agreements). Even switching to the *optimal* settings of a Learned Deviator tuned for a population of Sanctioning agents only yields a very small improvement of less than 0.3% in absolute winrate, to a winrate of less than 14.6%.

Hence, in a population of $n = 7$ Sanctioning agents, none of the agents can gain more than a 0.3% improvement in winrate by switching to another strategy within the space of the agents considered in this work and all settings for the Learned Deviator. In game theoretic terminology, this means that this is a ϵ-Nash equilibrium (a near-equilibrium), for $\epsilon = 0.3\%$.

When playing with agents that have stochastic policies, confidently determining the optimal course of action requires large amounts of data, in the form of simulated games. Hence, learning agents who are considering modifying their behavior face a demanding problem in terms of data and compute. Experimenting with their behavioral parameters by playing against other agents is costly not just in terms of compute, but also due to potentially losing games when switching to sub-optimal parameters. Such experimentation leads to a noisy estimate of winrate, which means that unless a change in behavior yields significant benefits it would be hard to notice with limited data.

In a population of Sanctioning agents, changing the behavior to deviating a large proportion of turns (e.g. to a Simple of Conditional Deviator, or thresholds t_ϕ, t_ψ that result in being willing to deviate in many turns) causes a significant reduction in winrate that is easy to

spot with limited data. In contrast switching to the optimal Learned Deviator thresholds only results in a very small improvement in winrate that requires large amounts of data to detect. Both human and various types of artificial agents exhibit risk aversion or a status-quo bias [41, 42, 43, 44], meaning that they only modify their behavior or decision from their current policy if they are confident such a change would result in a significantly higher utility (or if the risk of not achieving an improvement in utility is sufficiently low). When agents have even a slight status-quo bias, the Sanctioning agent population behavior is stable as the maximal gain agents can gain from modifying their behavior within the space we consider is $\epsilon = 0.3\%$ which is a very small advantage. Hence, this provides an account of how a population of Sanctioning agents with a status-quo bias may be capable of supporting honest cooperation.

11.2 Near-Stability of Mostly-Honest Communication

Section 11.1 discussed the near-equilibrium of Sanctioning agents. As Sanctioning agents do not themselves deviate from agreement, that equilibrium has completely truthful communication. However, as we saw, there was still a small incentive to adopt a Learned Deviator behavior in that equilibrium, so here we consider whether there exists an ϵ-Nash based on the Learned Deviator with an even smaller ϵ.

We consider a population of agents that mix the behaviors of Sanctioning agents and Learned Deviator agents. We call these agents Combined agents, as one the one hand they deviate from contracts under the same threshold settings t_ϕ, t_ψ as the Learned Deviator, but on the other hand they sanction other agents who deviate from agreements, similarly to the Sanctioning agents.

We consider a population comprised of Combined agents, and two ways in which a Combined agent in this population might change its agent type: by changing its deviation parameters, or by removing its sanctioning, leaving only the Binary defense (of refusing future communication with the deviator).

Changing the deviation parameters is explored in Figure 8. As Combined Deviators (with the same threshold parameters t_ϕ, t_ψ as the Learned Deviator) do not break agreements in most games, the relative performances of different deviator settings in Figure 8 are similar to those in the Section on the empirical analysis of Defensive Agents; the highest point estimate for the relative winrate is 101.3%, corresponding to an absolute winrate of $\frac{101.3\%}{101.3\%+6} = 14.44\%$.

Using data from over 150,000 games, we find that an agent that stops sanctioning achieves a winrate of 100.1% [99.8%, 100.5%] relative to that of Combined agents with the Mutual Proposal protocol. Similarly, with the Propose-Choose protocol, ceasing to sanction yields a winrate of 100.2% [99.9%, 100.4%] relative to the Combined agents. The upper end of these confidence intervals represents an absolute winrate of at most $\frac{100.5\%}{100.5\%+6} = 14.35\%$. Again, a reasonable explanation for these behavior changes having almost no impact on winrates is that the change in strategy only affects the agent's behavior in a small minority of games where others deviate against the agent; even within those games, the agent behaves differently

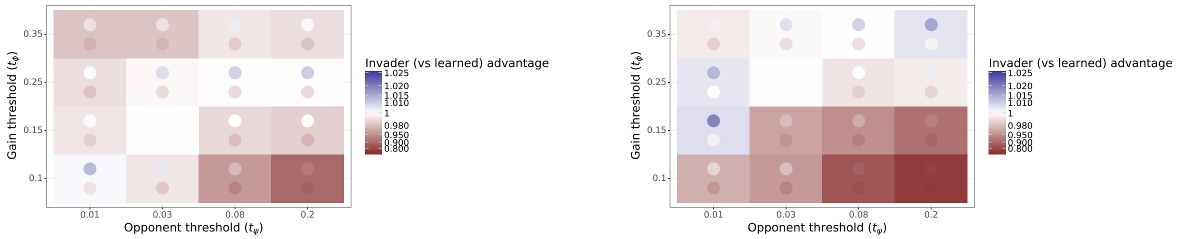

Supplementary Figure 8: Relative winrates of alternate Combined Agents that change their deviation parameters, faced with 6 unaltered Combined Agents; each based on over 20,000 games. (Left: Mutual-Proposal, Right:Propose-Choose)

only once the deviation has taken place, which tends to be near the end of the game.

Another possible strategy change is switching to an agent type that deviates much more frequently, such as becoming a Simple or Conditional Deviator. However, doing so results in a large decrease in winrate due to the sanctioning behavior of other agents in the population.

To summarize, given a population of Combined agents, no behavior change can achieve a winrate improvement greater than 0.2%, so we conclude that within our space of agent types, this strategy profile forms an ϵ-equilibrium with $\epsilon = 0.2\%$.

Overall, the above results indicate that a population of Combined Agents (with parameter settings t_ϕ, t_ψ identical to the Learned Deviator) is at an even nearer equilibrium than the ϵ-Nash equilibrium of Section 11.1: no agent can unilaterally switch its behavior and gain more than a small improvement of $\epsilon = 0.2\%$ in absolute winrate. As opposed to that equilibrium, under this equilibrium all agents do deviate from agreements. However, due to the choice of the parameter thresholds t_ϕ, t_ψ, which is the same as for the Learned Deviator, deviations remain rare, and the vast majority of communication under this equilibrium is truthful.

11.3 The Decay of Communication and Cooperation

The near-equilibria in Section 11.1 and Section 11.2 result in a lot of cooperative behavior by the agents. However, they are only near-equilibria, meaning that some agents could achieve a small improvement by modifying their behavior. In this section we consider a gradual learning process through which cooperation may decay over time.

First, consider a population of Sanctioning agents. As discussed in Section 11.1 this is a near-equilibrium, but a Learned Deviator slightly outperforms the Sanctioning agents in such a population. We also note that the performance of the Combined Agent from Section 11.2 against a population of Sanctioning agents is the same as that of the Learned Deviator: it uses the same thresholds t_ϕ, t_ψ as the Learned Deviator and thus has the same deviation behavior, and as the remaining agents are Sanctioning ones they never deviate from agreements and thus never trigger the Combined Agent's sanctioning behavior.

Learning agents, for instance those based on reinforcement learning algorithms, adapt their behavior based on past experience, by adjusting their parameters based on noisy estimates of

the impact of such behavioral changes on their utility. However, the slight advantage of the Learned Deviator means that given enough experience some learning algorithms may switch to a behavior more akin to the Combined Agent (or possibly that of a Learned Deviator). Now, suppose all the agents switch to the behavior of a Combined Agent.

When all agents are Combined Agents, Section 11.2 shows that ceasing the sanctioning behavior offers no more than a very small advantage, making it difficult to spot that advantage empirically, given the noisy signal obtained by measuring winrates over past games. However, by reasoning about the agent behavior we expect a priori that sanctioning is a costly behavior as compared to the Binary defense: rather than selecting actions so as to maximize its own value function, the sanctioning agent's behavior means they also attempt to lower the deviator's winrate (which is indirectly beneficial to any remaining players, other than the sanctioner and the deviator). Knowing this, Combined Agents have an incentive to switch to the Binary defense of stopping communication with others who have deviated from agreements with them, while retaining their own parametrized deviation behavior. In other words, learning may result in a population of agents who themselves deviate from contracts (with the same parameters as the Learned Deviators), and only cease communicating with other deviators rather than actively sanctioning them. We refer to these agents as *Initial Binary Deviators*.

In some infinitely repeated games, such as Iterated Prisoner's Dilemma, certain cooperative strategies are fully stable [21, 27, 28]. In contrast, our setting of Diplomacy is a non-repeated game with many players. Diplomacy is ultimately a zero-sum game where some agents win at the expense of others, making alliances difficult to maintain in the long term. This makes it a particularly challenging environment for achieving rational cooperation. Given a population of Initial Binary Deviators, this raises the question of whether the defense of ceasing communication can deter deviations robustly enough so as to avoid a further degradation of cooperative behavior (where we view deviation from past agreements as uncooperative behavior).

To understand this question better, it is worth noting a key difference between Binary and Sanctioning defenses: that sanctioning is mostly *defender deviation-independent*, while the binary defense is *defender deviation-dependent*. What we mean by this is that if a population uses a Sanctioning defense, and increases its level of deviation, the defense continues to significantly disincentivize deviation on the part of a single agent. On the other hand, if the population uses a Binary defense then the incentive not to deviate arises only from the potential future cooperation gains that are lost if the defense is triggered; as a result, if the population increases its level of deviation, then those potential cooperation gains proportionately decrease, and so does the disincentive for an agent to deviate. The consequence of this difference is that while the Combined agents population of Section 11.2 did not create a clear incentive to increase the level of deviation further beyond that of the Learned Deviator (as seen in Figure 8), with the Binary defense we may see agents learning to deviate more frequently.

Under the Mutual-Proposal protocol, the empirical results show that even against 6 Binary

Agents, who never deviate from agreements, a single Conditional Deviator has an advantage. Our Initial Binary Deviators exhibit the same reaction to others deviating from agreements, ceasing to communicate with deviators. Indeed, we find that a Conditional Deviator also outperforms Initial Binary Deviators when playing against 6 Initial Binary Deviators (with a relative winrate of 107%), meaning that a learning population would gradually shift its deviation parameters until deviation is predominant. At that point a Simple Deviator (that selects its action according to a non-negotiating policy) may actually gain an advantage over the Conditional Deviators, because the latter selects its deviating action according to an incorrect assumption that its counterpart will not deviate, while the former would not make any such incorrect assumptions.

In contrast, as we show in our empirical analysis for the Deviator Agents, under the Propose-Choose protocol a Conditional Deviator does not gain an advantage against 6 Binary Negotiators. Hence, we consider a range of Learned Deviator parameters t_ϕ, t_ψ seeking the most favorable ones for a perturbed Binary Deviator playing against 6 Binary Negotiators. In other words, we check whether within a population of Binary Negotiators one of the agents would improve its winrate by modifying its deviator parameters t_ϕ, t_ψ to deviate more (i.e. lower t_ϕ, higher t_ψ) than the Learned Deviator. The winrate advantage results are shown in Figure 9.

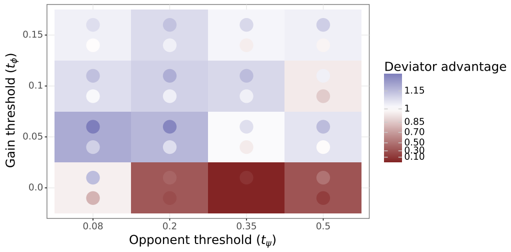

Supplementary Figure 9: Learned Deviator performance against 6 Binary agents with the Propose-Choose protocol. Parameters considered favor deviation more than in previous sections, because that yields higher performance against honest defenders. Higher deviation rates are towards the bottom right.

Figure 9 shows that for the Propose-Choose protocol, agents facing a population of Binary Negotiators obtain the greatest advantage by adopting deviation parameters $(t_\phi, t_\psi) = (0.05, 0.08)$, which are looser (deviate more) than the ones for the Learned Deviator; $(t_\phi, t_\psi) = (0.05, 0.2)$ is comparably strong, with no statistically significant difference from $(0.05, 0.08)$. These parameters result in never deviating in, respectively, 71% or 59% of games. In additional experiments we have verified that an agent with parameters $(0.05, 0.5)$ (which never deviates in 57% of games versus 6 non-deviating negotiators) has a winrate ratio of 1.03 [1.00, 1.06] over 6 agents with deviation parameters $(0.05, 0.2)$, and that a Conditional Deviator has a winrate ratio of 0.90 [0.83, 0.97] versus 6 agents with parameters $(0.05, 0.5)$. This suggests a near-equilibrium with parameters $(0.05, 0.5)$, in which communication and cooperative

behavior are still retained, despite a substantial increase in the deviation rate.

While for this case there is a near-equilibrium of Binary Deviators that retains cooperative behavior, we cannot rule out that careful measurement would show benefit to decreasing t_ϕ gradually to 0. Also, a backwards induction argument suggests that if we modify Learned Deviators to always deviate after a certain turn number, there may always be a benefit to reducing that deviation turn number, until it reaches 0.

The results in this section demonstrate how a gradual learning process can eventually lead to uncooperative outcomes, even when starting with the near-equilibrium of Section 11.1 that has truthful communication and significant cooperation. The initial near-equilibrium applies sanctioning behavior to deter deviations, and is indeed an ϵ-Nash (for $\epsilon = 0.3\%$), meaning that the incentives to modify the agent behavior are small and require many games to detect. Further, the process we described requires multiple such learning steps to gradually erode cooperation. Nonetheless, we feel that these results highlight the need to examine complementary mechanisms to deter uncooperative behaviors such as deviating from agreements. One avenue for future research is to employ trust and reputation systems [45, 46]. Under such a solution, agents could keep track and communicate regarding the behavior of others in the previous turns in the game or over multiple games. In Diplomacy one might implement this by modifying the protocol to allow agents to communicate their beliefs regarding the behavior of others. For example, a protocol could allow an agent to report to a third party that a peer has deviated from an agreement. Agents could then reason about the trustworthiness of a peer given the reports of others, and only agree to contracts with them if they deem them to be trustworthy enough.

More generally, human participants clearly do communicate and cooperate effectively in Diplomacy despite the incentive to deviate from agreements. An open question for future work is to better understand what drives this human cooperation. Do human participants assume a longer time horizon for cooperation, across multiple games? Does experience in other domains push them to avoid deviating from agreements? Do skilled players rely on increasingly cautious deals that rationally manage the downside risk of deviating peers? Answering such questions for human participants could go hand-in-hand with implementing similar mechanisms for supporting cooperation between artificial agents.

Supplementary Note 12 Detailed Code Listings

Data and code availability: the raw data for producing the images is available in a separate Excel file (submitted along this paper). The experiments are based on the algorithms contained in the Methods section, which are presented here in more detail using Python-style code snippets. These depend on the base No-Press Diplomacy policy and value functions [7], which are available at `https://github.com/deepmind/diplomacy`.

The Methods section in the main paper provided pseudo-code for the agent algorithms used in the experiments in Section 2 (Results). To aid clarity and reproducibility we now

provide more detailed Python-style code snippets for these algorithms as well.

Listing 1: Restriction Simulation Sampling

```python
def rss(state,
        player: int,
        peer_policy_fn,
        value_fn,
        num_action_profiles: int,
        contracts: Sequence[Tuple[Sequence[UnitAction], Sequence[UnitAction]]],
        defense=False,
        alpha=0.):
  offer_deal = [False] * NUM_PLAYERS
  no_deal_base_profiles = peer_policy_fn(num_action_profiles, state)  # [n_a_p, 7]
  values_no_deal = [
      estimate_values(value_fn, [base_profile], state, [{}])
      for base_profile in no_deal_base_profiles
  ]
  sanction_adjust = state.sanction_coefs(player, alpha).dot
  sanction_value_no_deal = sanction_adjust(np.mean(values_no_deal, axis=0))
  for partner in range(NUM_PLAYERS):
    if partner == player or (defense and state.deviated(partner, vs=player)):
      continue
    deal_legal_actions = list(state.legal_actions)
    deal_legal_actions[player], deal_legal_actions[partner] = contracts[partner]
    if not any(
        set(profile[partner]) - set(contracts[partner][1])
        for profile in no_deal_base_profiles):
      continue  # Reject deal that may not constrain peer.
    deal_values = []
    for profile, values in zip(no_deal_base_profiles, values_no_deal):
      resample_for = []
      if set(profile[player]) - set(deal_legal_actions[player]):
        resample_for.append(player)
      if set(profile[partner]) - set(deal_legal_actions[partner]):
        resample_for.append(partner)
      if not resample_for:
        deal_values.append(values)
        continue
      peace_actions, = peer_policy_fn(
          1, state, players=resample_for, legal_actions=deal_legal_actions)
      deal_values.extend(
          estimate_values(value_fn, [profile], state,
                          [dict(zip(resample_for, peace_actions))]))
    sanction_value_deal = sanction_adjust(np.mean(deal_values, axis=0))
    if sanction_value_deal > sanction_value_no_deal:
      offer_deal[partner] = True
  return offer_deal
```

Listing 2: Nash Bargaining Score

```python
Deal = Mapping[int, Sequence[UnitAction]]
class DealCandidate(NamedTuple):
  value: float
  partner: int
  deal: Deal
  raw_values: List[float]

  def nash_score(self, player_id, batna):
    advantage_player = self.values[player_id] - batna[player_id, self.partner]
    advantage_partner = self.values[partner] - batna[self.partner, player_id]
    return max(0, advantage_player) * max(0, advantage_partner)
```

Listing 3: BATNA Update by Internal Dynamic Bargaining Simulation

```
Values = np.ndarray  # [NUM_PLAYERS]
BATNAs = Mapping[Tuple[int, int], Values]
DealValues = Mapping[Tuple[int, int], Sequence[Values]]
Deals = Mapping[Tuple[int, int], Sequence[Deal]]
def mbds_bargain(batna: BATNAs, sbr_batna: BATNAs, deal_values: DealValues,
                 active_players: Sequence[int], batna_damping: float) -> BATNAs:
  new_batna = sbr_batna.copy()
  for (i, j), values in deal_values.items():
    adv_i, adv_j = values[:, i] - batna[i, j], values[:, j] - batna[j, i]
    prod_advantage = np.maximum(0, adv_i) * np.maximum(0, adv_j)
    if prod_advantage.any():
      values = values[prod_advantage.argmax()]
      for k in active_players - {i, j}:
        new_batna[i, k] = max(new_batna[i, k], values[i])
        new_batna[j, k] = max(new_batna[j, k], values[j])
  for slots in batna.keys():
    new_batna[slots] = batna[slots] + (1 - batna_damping) * (
        new_batna.get(slots, batna[slots]) - batna[slots])
  return new_batna
```

Listing 4: Mutually Beneficial Deal Sampling Proposal

```
def mbds_propose(state, player: int, candidate_policy_fn, peer_policy_fn,
                 value_fn, num_action_profiles: int,
                 num_sampled_candidates: int, num_deal_candidates: int,
                 partner_value_weight: float, num_bargain_rounds: int,
                 bargain_damping_factor: float):
  candidates = candidate_policy_fn(num_sampled_candidates, state)  # [7, n_s_c]
  base_profiles = peer_policy_fn(num_action_profiles, state)  # [n_a_p, 7]

  estimate_values_fn = functools.partial(estimate_values, value_fn,
                                         base_profiles, state)
  (player_candidates, unilateral_values) = (
      compute_player_candidates_and_unilateral_values(
          candidates, estimate_values_fn))
  unilateral_values_np = [np.asarray(x) for x in unilateral_values]
  player_candidates_np = [np.asarray(x) for x in player_candidates]

  sbr_pair_values = compute_sbr_pair_values(unilateral_values_np,
                                            player_candidates_np,
                                            estimate_values_fn)
  deals, deal_values = compute_deals_and_values(
      unilateral_values_np, player_candidates_np, sbr_pair_values,
      num_deal_candidates, partner_value_weight, estimate_values_fn)
  batna = compute_batna(
      deal_values, {i for i, c in enumerate(player_candidates_np) if c.size},
      sbr_pair_values, bargain_damping_factor, num_bargain_rounds)
  offers = compute_offers(player, deals, deal_values, batna)
  return offers, batna, sbr_pair_values


def compute_player_candidates_and_unilateral_values(
    candidates, estimate_values_fn
) -> Tuple[Sequence[Sequence[Sequence[UnitAction]]], Sequence[Sequence[float]]]:
  all_candidates = []
  for player_index, actions in enumerate(candidates):
    all_candidates.extend([{player_index: c} for c in actions])

  player_candidates = [[] for _ in range(NUM_PLAYERS)]
  unilateral_values = [[] for _ in range(NUM_PLAYERS)]
```

```
  for candidate, values in estimate_values_fn(all_candidates):
    (player_index, candidate), = candidate.items()
    unilateral_values[player_index].append(values)
    player_candidates[player_index].append(candidate)
  return player_candidates, unilateral_values


def compute_sbr_pair_values(
    unilateral_values, player_candidates,
    estimate_values_fn) -> Mapping[Tuple[int, int], np.ndarray]:
  """Returns values for SBRs faced off against each other."""
  active_slots = {i for i, c in enumerate(player_candidates) if c.size}
  sbr_pair_candidates = []
  for i in active_slots:
    for j in set(range(i)) & active_slots:
      sbr_pair_candidates.append({
          i: player_candidates[i][unilateral_values[i][:, i].argmax()],
          j: player_candidates[j][unilateral_values[j][:, j].argmax()]
      })

  sbr_pair_values = {}
  for candidate, values in estimate_values_fn(sbr_pair_candidates):
    slot1, slot2 = candidate.keys()
    sbr_pair_values[slot1, slot2] = sbr_pair_values[slot2, slot1] = values
  return sbr_pair_values


def compute_deals_and_values(
    unilateral_values, player_candidates, sbr_pair_values,
    num_slot_offer_candidates, partner_weight, estimate_values_fn
) -> Tuple[Deals, DealValues]:
  active_slots = {i for i, c in enumerate(player_candidates) if c.size}
  deal_candidates = {}
  for slot in active_slots:
    for partner in active_slots - {slot}:
      if (slot, partner) not in sbr_pair_values:
        continue
      pair_values = sbr_pair_values[slot, partner]
      uni_values = unilateral_values[slot]
      criterion = (
          uni_values[:, slot] / pair_values[slot] +
          partner_weight * uni_values[:, partner] / pair_values[partner])
      indices = criterion.argsort()[-num_slot_offer_candidates:]
      deal_candidates[slot, partner] = [
          tuple(cand) for cand in player_candidates[slot][indices]
      ]

  all_deals = []
  deals = collections.defaultdict(list)
  deal_values = collections.defaultdict(list)
  for i in active_slots:
    for j in active_slots & set(range(i)):
      deal_values[i, j] = deal_values[j, i]
      dij = deals[i, j] = deals[j, i]
      if (i, j) not in deal_candidates:
        continue
      for deal_i in deal_candidates[i, j]:
        dij += [{i: deal_i, j: deal_j} for deal_j in deal_candidates[j, i]]
      all_deals += dij
  for candidates, values in estimate_values_fn(all_deals):
    deal_values[tuple(candidates.keys())].append(np.ndarray(values))
  return deals, deal_values
```

```python


def compute_batna(deal_values, active_players, sbr_pair_values, batna_damping,
                  num_bargain_rounds) -> BATNAs:
  sbr_batna = {}
  for (slot, partner), pair_values in sbr_pair_values.items():
    sbr_batna[slot, partner] = pair_values[slot]
  batna = sbr_batna.copy()
  for _ in range(num_bargain_rounds):
    batna = mbds_bargain(batna, sbr_batna, deal_values, active_players,
                         batna_damping)
  return batna


def compute_offers(player, deals, deal_values, batna) -> Sequence[Deal]:
  offers = []
  for (i, j), values in deal_values.items():
    values = np.stack(values)
    if i == player and len(values):
      adv_i, adv_j = values[:, i] - batna[i, j], values[:, j] - batna[j, i]
      prod_advantage = np.maximum(0, adv_i) * np.maximum(0, adv_j)
      if prod_advantage.any():
        deal = deals[i, j][prod_advantage.argmax()]
        offers.append(deal)
  return offers
```

Listing 5: Mutually Beneficial Deal Sampling Choice

```python
def mbds_choose(state,
                player,
                base_profiles,
                value_fn,
                sbr_pair_values: Mapping[Tuple[int, int], np.ndarray],
                batna: Mapping[Tuple[int, int], np.ndarray],
                deals_sent: Sequence[Optional[Deal]],
                deals_rcvd: Sequence[Optional[Deal]],
                defense=False,
                alpha=0) -> Sequence[Deal]:
  deals = [deal for deal in deals_sent + deals_rcvd if deal is not None]
  deals_with: List[List[Optional[DealCandidate]]] = [
      [] for partner in range(NUM_PLAYERS)
  ]
  sanction_adjust = state.sanction_coefs(player, alpha).dot
  for deal, values in estimate_values(value_fn, base_profiles, deals, state):
    partner, = set(deal.keys()) - {player}
    if defense and (state.deviated(partner, vs=player) or
                    state.deviated(player, vs=partner)):
      continue
    deals_with[partner].append(
        DealCandidate(sanction_adjust(values), partner, deal, values))
  both_acceptable = [False] * NUM_PLAYERS
  best_deal = DealCandidate(-np.inf, -1, {}, [])
  no_deal_value_vs = [
      sanction_adjust(sbr_pair_values[player, partner])
      for partner in range(NUM_PLAYERS)
  ]
  nash_score_fn = lambda candidate: candidate.nash_score(player, batna)

  # Identify Best Fair Deal
  for partner, partner_deals in enumerate(deals_with):
    if partner_deals:
      fairest = max(partner_deals, key=nash_score_fn)
```

```python
      if (fairest.value > no_deal_value_vs[partner] and nash_score_fn(fairest)):
        best_deal = max(best_deal, fairest)

      both_acceptable[partner] = (
          (min(partner_deals).value > no_deal_value_vs[partner]) and
          len(partner_deals) == 2)

  def retval_if_best(deal_candidate):
    if not both_acceptable[deal_candidate.partner]:
      return [deal_candidate.deal]
    deals_with[deal_candidate.partner].sort(key=lambda x: x != deal_candidate)
    return [candidate.deal for candidate in deals_with[deal_candidate.partner]]

  if best_deal.value > -np.inf:
    return retval_if_best(best_deal)

  # Identify Best Favourable Deal
  for partner, partner_deals in enumerate(deals_with):
    for deal_candidate in partner_deals:
      if deal_candidate.value > no_deal_value_vs[partner]:
        best_deal = max(best_deal, deal_candidate)

  if best_deal.value > -np.inf:
    return retval_if_best(best_deal)
  return []
```

Listing 6: Peace Definition

```python
def peaceful_actions(state, peace_deals: Sequence[Sequence[int]]):
  # Identify whose unit is in the province.
  provinces_to_unit_powers = {}
  for player in range(NUM_PLAYERS):
    for action in state.legal_actions[player]:
      order, (p_1, _), (p_2, _), (p_3, _) = utils.action_breakdown(action)
      provinces_to_unit_powers[p_1] = player

  retval = list(state.legal_actions)
  for player in range(NUM_PLAYERS):
    if not peace_deals[player]:
      continue
    retval[player] = []
    for action in state.legal_actions[player]:
      order, _, (p_2, _), (p_3, _) = utils.action_breakdown(action)
      if p_2 in utils.all_sc_provinces(state.board):
        sc_owner = utils.sc_owner(
            utils.area_from_province_id_and_area_index(p_2, 0), state.board)
        if sc_owner in peace_deals[player]:
          if (order in (utils.MOVE_TO, utils.CONVOY_TO) or
              (order in (utils.SUPPORT_MOVE_TO, utils.CONVOY) and
               # p_3 contains the supported/convoyed unit
               provinces_to_unit_powers[p_3] != sc_owner) or
              (order == utils.SUPPORT_HOLD and
               # p_2 contains the supporting holding unit
               provinces_to_unit_powers[p_2] != sc_owner)):
            continue
      if provinces_to_unit_powers.get(p_2) in peace_deals[player]:
        if (order in (utils.MOVE_TO, utils.CONVOY_TO) or
            (order in (utils.SUPPORT_MOVE_TO, utils.CONVOY) and
             # p_2 is the area being convoyed or support-moved to
             provinces_to_unit_powers[p_2] != provinces_to_unit_powers[p_3])):
          continue
      # Checked all the ways of breaking peace deals, didn't find any.
```

```
      retval[player].append(action)
  return retval
```

Listing 7: Propose-Choose Deal Selection

```
def apply_chosen_deals(state, chosen: Sequence[List[Deal]]) -> Sequence[Deal]:
  retval = dict(enumerate(state.legal_actions))
  for power in range(NUM_PLAYERS):
    for partner in range(power):
      if not (partner in (chosen[power] or [{}])[0] and
              power in (chosen[partner] or [{}])[0]):
        continue
      assert all({partner, power} == set(deal.keys())
                 for deal in chosen[power] + chosen[partner])
      if chosen[power][0] == chosen[partner][0]:
        retval.update(chosen[power][0])
      elif chosen[power][1:]:
        if chosen[partner][1:]:
          retval.update(random.choice(chosen[power]))
        else:
          retval.update(chosen[partner][0])
      elif chosen[partner][1:]:
        retval.update(chosen[power][0])
  return list(retval.values())
```

Supplementary References

[1] A. Calhamer, “Diplomacy. Board Game,” *Avalon Hill*, 1959.

[2] P. Paquette, Y. Lu, S. S. Bocco, M. Smith, O.-G. Satya, J. K. Kummerfeld, J. Pineau, S. Singh, and A. C. Courville, “No-press Diplomacy: Modeling multi-agent gameplay,” in *Advances in Neural Information Processing Systems (NIPS)*, pp. 4476–4487, 2019.

[3] J. Farrell and M. Rabin, “Cheap talk,” *Journal of Economic Perspectives*, vol. 10, no. 3, pp. 103–118, 1996.

[4] H. Hu, A. Lerer, A. Peysakhovich, and J. Foerster, ““other-play” for zero-shot coordination,” in *International Conference on Machine Learning*, pp. 4399–4410, PMLR, 2020.

[5] OpenAI, “Better language models and their implications,” *San Francisco: OpenAI*, 2019.

[6] W. Hoeffding, “Probability inequalities for sums of bounded random variables,” in *The collected works of Wassily Hoeffding*, pp. 409–426, Springer, 1994.

[7] T. Anthony, T. Eccles, A. Tacchetti, J. Kramár, I. Gemp, T. C. Hudson, N. Porcel, M. Lanctot, J. Pérolat, R. Everett, *et al.*, “Learning to play no-press Diplomacy with best response policy iteration,” in *Advances in Neural Information Processing Systems (NIPS)*, 2020.

[8] A. Rose, D. Normal, and H. Williams, "Diplomacy artificial intelligence development environment," *DAIDE*, 2002. http://www.daide.org.uk.

[9] J. S. Rosenschein, "Rational interaction: Cooperation among intelligent agents," tech. rep., Stanford Univ., CA (USA), 1986.

[10] J. S. Rosenschein and G. Zlotkin, *Rules of encounter: designing conventions for automated negotiation among computers*. MIT press, 1994.

[11] S. Kraus, E. Ephrati, and D. Lehmann, "Negotiation in a non-cooperative environment," *Journal of Experimental & Theoretical Artificial Intelligence*, vol. 3, no. 4, pp. 255–281, 1994.

[12] S. Kraus and D. Lehmann, "Designing and building a negotiating automated agent," *Computational Intelligence*, vol. 11, no. 1, pp. 132–171, 1995.

[13] T. Sandholm, V. R. Lesser, *et al.*, "Issues in automated negotiation and electronic commerce: Extending the contract net framework," in *ICMAS*, vol. 95, pp. 12–14, 1995.

[14] N. R. Jennings, K. Sycara, and M. Wooldridge, "A roadmap of agent research and development," *Autonomous agents and multi-agent systems*, vol. 1, no. 1, pp. 7–38, 1998.

[15] P. Faratin, C. Sierra, and N. R. Jennings, "Negotiation decision functions for autonomous agents," *Robotics and Autonomous Systems*, vol. 24, no. 3-4, pp. 159–182, 1998.

[16] M. Beer, M. d'Inverno, M. Luck, N. Jennings, C. Preist, and M. Schroeder, "Negotiation in multi-agent systems," *The Knowledge Engineering Review*, vol. 14, no. 3, pp. 285–289, 1999.

[17] S. Kraus and R. C. Arkin, *Strategic negotiation in multiagent environments*. MIT press, 2001.

[18] N. R. Jennings, P. Faratin, A. R. Lomuscio, S. Parsons, M. J. Wooldridge, and C. Sierra, "Automated negotiation: Prospects, methods and challenges," *Group Decision And Negotiation*, vol. 10, no. 2, pp. 199–215, 2001.

[19] S. Fatima, S. Kraus, and M. Wooldridge, *Principles of automated negotiation*. Cambridge University Press, 2014.

[20] U. Kiruthika, T. S. Somasundaram, and S. Raja, "Lifecycle model of a negotiation agent: A survey of automated negotiation techniques," *Group Decision and Negotiation*, vol. 29, no. 6, pp. 1239–1262, 2020.

[21] R. Axelrod and W. D. Hamilton, "The evolution of cooperation," *Science*, vol. 211, no. 4489, pp. 1390–1396, 1981.

[22] P. D. Bó, "Cooperation under the shadow of the future: experimental evidence from infinitely repeated games," *American economic review*, vol. 95, no. 5, pp. 1591–1604, 2005.

[23] J. Shalev, "Nonzero-sum two-person repeated games with incomplete information and known-own payoffs," *Games and Economic Behavior*, vol. 7, no. 2, pp. 246–259, 1994.

[24] E. Israeli, "Sowing doubt optimally in two-person repeated games," *Games and Economic Behavior*, vol. 28, no. 2, pp. 203–216, 1999.

[25] V. Anderhub, D. Engelmann, and W. Güth, "An experimental study of the repeated trust game with incomplete information," *Journal of Economic Behavior & Organization*, vol. 48, no. 2, pp. 197–216, 2002.

[26] J. Bracht and N. Feltovich, "Whatever you say, your reputation precedes you: Observation and cheap talk in the trust game," *Journal of public economics*, vol. 93, no. 9-10, pp. 1036–1044, 2009.

[27] D. Fudenberg and E. Maskin, "The folk theorem in repeated games with discounting or with incomplete information," in *A long-run collaboration on long-run games*, pp. 209–230, World Scientific, 2009.

[28] J. Hofbauer and K. Sigmund, "Evolutionary game dynamics," *Bulletin of the American Mathematical Society*, vol. 40, no. 4, pp. 479–519, 2003.

[29] M. J. Osborne and A. Rubinstein, *A course in game theory.* MIT press, 1994.

[30] V. Conitzer and T. Sandholm, "Complexity results about nash equilibria," *arXiv preprint cs/0205074*, 2002.

[31] C. Daskalakis, P. W. Goldberg, and C. H. Papadimitriou, "The complexity of computing a nash equilibrium," *SIAM Journal on Computing*, vol. 39, no. 1, pp. 195–259, 2009.

[32] S. Kraus and D. Lehmann, "Diplomat, an agent in a multi agent environment: An overview," in *Seventh Annual International Phoenix Conference on Computers an Communications. 1988 Conference Proceedings*, pp. 434–438, IEEE, 1988.

[33] J. Gray, A. Lerer, A. Bakhtin, and N. Brown, "Human-level performance in no-press diplomacy via equilibrium search," in *International Conference on Learning Representations (ICLR)*, 2020.

[34] G. Tesauro, "TD-Gammon, a self-teaching Backgammon program, achieves master-level play," *Neural Computation*, vol. 6, no. 2, pp. 215–219, 1994.

[35] D. Silver, A. Huang, C. J. Maddison, A. Guez, L. Sifre, G. Van Den Driessche, J. Schrittwieser, I. Antonoglou, V. Panneershelvam, M. Lanctot, *et al.*, "Mastering the game of Go with deep neural networks and tree search," *Nature*, vol. 529, no. 7587, p. 484, 2016.

[36] D. Silver, J. Schrittwieser, K. Simonyan, I. Antonoglou, A. Huang, A. Guez, T. Hubert, L. Baker, M. Lai, A. Bolton, *et al.*, "Mastering the game of Go without human knowledge," *Nature*, vol. 550, no. 7676, pp. 354–359, 2017.

[37] K. Binmore, A. Rubinstein, and A. Wolinsky, "The Nash bargaining solution in economic modelling," *The RAND Journal of Economics*, pp. 176–188, 1986.

[38] E. H. Gerding, D. D. B. van Bragt, and J. A. La Poutré, *Scientific approaches and techniques for negotiation: a game theoretic and artificial intelligence perspective*. Citeseer, 2000.

[39] J. F. Nash *et al.*, "Equilibrium points in n-person games," *Proceedings of the National Academy of Sciences*, vol. 36, no. 1, pp. 48–49, 1950.

[40] A. Blum and Y. Mansour, "Learning, regret minimization, and equilibria," in *Algorithmic Game Theory*, ch. 4, Cambridge University Press, 2007.

[41] M. Heger, "Consideration of risk in reinforcement learning," in *Machine Learning Proceedings 1994*, pp. 105–111, Elsevier, 1994.

[42] W. Samuelson and R. Zeckhauser, "Status quo bias in decision making," *Journal of risk and uncertainty*, vol. 1, no. 1, pp. 7–59, 1988.

[43] Y. Masatlioglu and E. A. Ok, "Rational choice with status quo bias," *Journal of economic theory*, vol. 121, no. 1, pp. 1–29, 2005.

[44] S. Obraztsova, E. Markakis, and D. R. Thompson, "Plurality voting with truth-biased agents," in *International Symposium on Algorithmic Game Theory*, pp. 26–37, Springer, 2013.

[45] C. Sierra and J. Debenham, "An information-based model for trust," in *Autonomous Agents and Multiagent Systems (AAMAS)*, pp. 497–504, 2005.

[46] J. Sabater and C. Sierra, "Review on computational trust and reputation models," *Artificial Intelligence Review*, vol. 24, no. 1, pp. 33–60, 2005.
